# Multi-Objective Evaluation of Wearable-Assisted Radar-Only Rehabilitation Monitoring

**DOI:** 10.3390/s26216970

**Published:** 2026-11-04

**Authors:** Hrithik Majumdar Shibu, Seyed-Ali Sadegh-Zadeh, Delaram Jarchi

**Affiliations:** 1 School of Computer Science and Electronic Engineering, University of Essex, Colchester CO4 3SQ, UK; hrithik11804064@gmail.com; 2 Department of Computing, University of Staffordshire, Stoke-on-Trent ST4 2DE, UK; ali.sadegh-zadeh@staffs.ac.uk

**Keywords:** mmWave radar, rehabilitation monitoring, human activity recognition, knowledge distillation, inertial measurement unit, corruption robustness, calibration, subject-independent evaluation

## Abstract

Millimetre-wave radar offers contactless exercise sensing, while wearable inertial measurement units (IMUs) can supply privileged training supervision without being required at inference. We study radar-only exercise recognition on the mRI dataset (20 healthy subjects; ten exercises) and distinguish descriptive historical architectures from matched RTPD-Net versus constrained fine-tuning (CFT) comparisons. Historical RTPD-Net, PARETO-RKD, and AURORA-RKD were trained using different budgets, seeds, or corruption settings and cannot establish a preferred method. For a fixed 40-segment test split, the original evaluation reported mean corrupted macro-F1 changing from 0.5332 to 0.6945; a paired re-evaluation of saved checkpoints, using 11 corrupted conditions and five realisations each, instead obtained 0.5370 versus 0.6374 (difference 0.1004). The subject-cluster bootstrap interval, conditional on the same four subjects, was [0.0683, 0.1283], but the exact two-sided subject-level prediction-swap test gave p=0.125 (four independent test subjects), so statistical significance is not established. Five independently generated subject-partition seeds (25 fully retrained folds) produced mean corrupted macro-F1 of 0.5375 for the fold-local RTPD reference versus 0.5449 for the validation-gated policy (mean difference 0.0074); fine-tuning was accepted in 5 of 25 folds. These results demonstrate that the validation gate usually retains the reference and that the observed adaptation gains are not established as broadly useful; they also indicate that robustness benefits are heterogeneous and that larger independent-subject evaluations are necessary.

## 1. Introduction

Home rehabilitation requires objective monitoring outside the clinic, yet the sensor that is most informative during model development may be unsuitable for continuous deployment. Cameras provide rich kinematic data but raise visibility, lighting, and privacy concerns. Wearable IMUs capture body dynamics directly but depend on attachment, charging, positioning, and continued user compliance. Millimetre-wave (mmWave) radar is contactless, works without visible imagery, and captures motion signatures under weak illumination, making it attractive for rehabilitation and assisted-living environments [1,2,3,4]. The central problem is whether a multimodal radar-IMU teacher can improve a radar-only student without requiring the wearable at inference. The practical objective is to identify which prescribed rehabilitation exercise a person is performing from radar measurements. Such recognition could support adherence monitoring and remote rehabilitation training outside the clinic while avoiding continuous camera recording or reliance on wearable sensors during deployment.

The public mRI dataset was designed for this multimodal setting, providing synchronised radar, RGB-D, and inertial recordings from 20 participants performing rehabilitation exercises [1]. Because mRI provides synchronised radar and IMU recordings, it enables the IMU to be used as privileged supervision during training while retaining radar-only inference at deployment. The practical question is not simply whether multimodal fusion is accurate, but whether privileged wearable information can improve a radar-only model that remains usable when the wearable is removed.

This deployment setting relates to learning using privileged information [5] and cross-modal KD, where a multimodal teacher guides a unimodal deployment model [6,7,8,9]. Existing work largely optimises recognition accuracy, multimodal fusion, or missing-modality tolerance separately. Recent radar rehabilitation studies demonstrate that non-optical sensing can recognise therapeutic movements, while recent radar–inertial HAR work shows that synchronous sensor fusion can improve activity discrimination [10,11]. However, these settings do not directly answer the deployment question studied here: how to exploit a wearable modality during training, remove it completely at inference, and still evaluate the resulting radar-only model under clean, corrupted, calibrated, and unseen-subject conditions.

The research gap is therefore methodological rather than merely architectural. There is limited evidence on radar-only rehabilitation recognition in which (i) wearable information is explicitly treated as privileged training supervision, (ii) corruption robustness and calibration are evaluated alongside clean recognition, (iii) subject-disjoint deployment decisions are made without test-set selection, and (iv) a failed adaptation can safely revert to a trusted radar-only anchor. The scientific novelty of this work is the combination of this privileged-sensing formulation with a multi-objective evidence hierarchy and a validation-gated constrained fine-tuning policy that does not change the deployment architecture. The proposed framework is intended to reveal when adaptation is beneficial and when retaining the original radar model is the safer choice, rather than to claim a universally dominant network.

This study asks three research questions (RQs): RQ1: Does privileged wearable supervision provide useful radar-only exercise-recognition signals? RQ2 (descriptive): What clean-recognition, corruption-retention, and calibration behaviours were observed in the successive historical method implementations, without asserting a comparative ranking? RQ3: When assessed on identical inputs and held-out subjects, does validation-gated constrained fine-tuning improve corrupted radar predictions while avoiding unacceptable clean-validation degradation? The primary hypothesis is not that one architecture is universally best, but that validation screening is necessary because the benefit of adaptation varies with the subject partition. 

The contributions are (i) a unified deployment analysis spanning standard KD, representation-level distillation, counterfactual robustness, quality-aware aggregation, and constrained adaptation; (ii) a validation-safe fine-tuning objective that anchors a trusted RTPD student while learning from corrupted radar views; (iii) fold-local subject-disjoint evaluation with training-only normalisation, fold-local SSL, teacher/student retraining, and automatic RTPD fallback; and (iv) an explicit evidence hierarchy separating descriptive historical comparisons from matched and fold-local claims. The work is a preclinical algorithmic study on healthy participants; it does not claim clinical effectiveness.

The remainder of the article is organised as follows. Section 2 reviews radar rehabilitation sensing, privileged/cross-modal distillation, and robustness and calibration. Section 3 defines the proposed training and deployment framework and the constrained fine-tuning policy. Section 4 describes the mRI acquisition hardware, subject splits, optimisation, corruptions, and statistical procedures. Section 5 reports the descriptive historical regimes, the matched constrained fine-tuning experiment, ablations, and fold-local subject-disjoint deployment results. Section 6 and Section 7 summarise the conclusions and limitations, respectively.

## 2. Background and Related Works

### 2.1. Radar-Based Rehabilitation and Human Activity Sensing

Radar-based human activity recognition (HAR) has progressed from handcrafted micro-Doppler features to spatio-temporal deep models. Radar is relevant to healthcare because it does not capture conventional optical imagery and does not require body-worn instrumentation [2,3,4,12]. The recent literature further emphasises radar’s role in unobtrusive health and telemedicine monitoring and the continuing shift toward richer point-cloud, time–frequency, and multi-feature radar representations [13,14,15]. The mmWave-Based Assistive Rehabilitation System (MARS) demonstrated mmWave sensing for assistive rehabilitation [2], while more recent work has used 3D mmWave point clouds for rehabilitation-oriented HAR and single-radar recognition of therapeutic upper-limb exercises [4,10]. These studies support the feasibility of non-camera rehabilitation sensing; however, they principally evaluate direct radar recognition rather than wearable-assisted training with radar-only inference.

Multimodal datasets such as mRI and MM-Fi demonstrate the value of synchronised radar, vision, and inertial measurements [1,16]. Recent radar–inertial HAR systems have also shown that complementary temporal information can be fused effectively when both modalities remain available at inference [11]. Taken together with recent radar-HAR surveys and robust lightweight radar models, this work highlights a current deployment trend toward privacy-preserving sensing that must remain reliable under practical signal variation [12,15]. That assumption differs from the present deployment setting. Here, the IMU is deliberately unavailable to the final model: it is used only to shape the radar representation during training. This distinction motivates evaluation not only of clean classification but also of whether the radar-only student remains reliable after the privileged modality is removed.

### 2.2. Cross-Modal Distillation with Missing or Privileged Modalities

KD transfers class distributions or intermediate representations from a teacher to a smaller or modality-restricted student [6]. Vision-to-sensor and skeleton-to-sensor distillation show that privileged modalities can improve wearable HAR while remaining absent at inference [7,8]. Incomplete modality KD similarly uses available modalities to train a deployable representation without imputing missing inputs [9]. Recent HAR studies extend this idea to multi-inertial teacher–student learning, compact wearable deployment, and uncertainty-aware distillation, reinforcing the value of transferring richer training-time information into a constrained inference model [17,18,19]. Multiscale distillation has further shown that intermediate-level transfer can improve multimodal HAR [20]. These works establish useful transfer mechanisms—soft logits, intermediate features, cross-modal structure, and uncertainty-aware supervision—but do not directly determine which transferred information remains reliable when the deployed sensor is corrupted or when the test subject is unseen. Our study reverses the common wearable-deployment setting: the wearable teaches while radar is deployed, and the resulting radar student is judged under multiple deployment objectives rather than clean accuracy alone.

### 2.3. Robustness, Calibration, and Safe Adaptation

Corruption mixtures and consistency regularisation improve common-corruption robustness [21]; group-DRO targets worst-group behaviour [22]; and supervised contrastive learning can stabilise class representations [23]. Recent radar-HAR work likewise places increasing emphasis on robustness under challenging sensing conditions and computationally efficient deployment [15]. Neural classifiers are also often miscalibrated, with temperature scaling and deep ensembles providing strong baselines [24,25]. Conformal prediction provides set-valued uncertainty estimates [26], while uncertainty-aware wearable-sensor distillation illustrates how reliability information can be incorporated directly into compact student learning [19]; L2-SP retains proximity to a trusted initial solution during transfer [27]. These techniques address different failure modes and can conflict: a robustness-oriented update may alter clean accuracy or calibration, while an ensemble can improve uncertainty estimates at additional inference cost. For rehabilitation monitoring, such trade-offs matter because sensor degradation and subject shift occur at deployment rather than during model development. The unresolved gap is therefore not the absence of any one technique but the lack of a radar-only deployment study that explicitly separates clean accuracy, corruption robustness, calibration, cross-subject generalisation, and validation-safe adaptation.

### 2.4. Method Provenance and Scope of Comparison

Standard KD and the cited robustness, calibration, and transfer-learning techniques are established methods from the literature. In particular, we follow Hinton et al. for logit knowledge distillation [6], Hendrycks et al. for corruption/consistency robustness [21], Sagawa et al. for group-robust optimisation [22], Guo et al. for calibration [24], Lakshminarayanan et al. for ensemble uncertainty [25], and Li et al. for L2-SP transfer regularisation [27]. In contrast, the labels RTPD-Net, PARETO-RKD, and AURORA-RKD in this manuscript denote successive methods developed by the authors within this study; they are not imported published baselines and therefore do not have external method citations. RTPD-Net is the authors’ reliability-aware temporal/prototype distillation formulation, PARETO-RKD is the authors’ corruption- and calibration-oriented extension with gradient-safe auxiliary routing, and AURORA-RKD is the authors’ quality-aware multi-branch aggregation framework. Because these variants were developed at different stages with different optimisation budgets and some different corruption settings, their historical results are used only to characterise operating regimes. The only controlled final comparison is the matched RTPD anchor versus constrained RTPD fine-tuning, for which the split, corruption suite, calibration procedure, and evaluation code are held fixed.

## 3. Methodology

### 3.1. Problem Formulation and Evidence Hierarchy

Let $x^{r}\in R^{T\times 14\times 14\times 5}$ denote a radar sequence. Each frame is the precomputed 14×14 mRI radar feature map, and the five channels store the *x*-, *y*-, and *z*-coordinate features, Doppler velocity, and return intensity provided by the released dataset [1,28]. Let $x^{i}\in R^{T\times 72}$ denote the synchronised IMU sequence: six wearable units each contribute 12 values (a flattened 3×3 orientation matrix and three acceleration components), yielding 72 features per frame. The exercise label is y∈{1,…,C}, with T=96 resampled frames and C=10 classes. During training, the multimodal teacher may access $\left(x^{r},x^{i}\right)$; deployment is strictly radar-only, with $f_{\theta }\left(x^{r}\right)$ producing the exercise class prediction.

Because the historical methods were not retrained with identical experimental budgets, RQ2 is strictly descriptive: no numerical "preferred method" is inferred from their reported rankings. Within-method result shifts across historical reruns are larger than the modest 0.0204 clean macro-F1 change in the original fixed-split RTPD/CFT comparison. 

**Figure 1:**
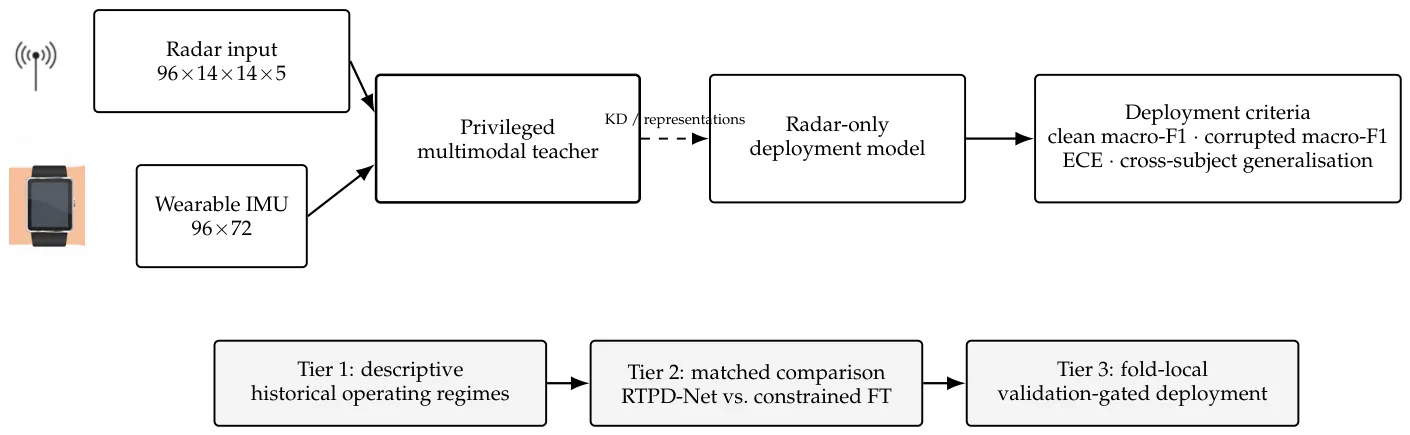
Wearable-assisted training, radar-only deployment, and the evidence hierarchy used to distinguish unmatched historical results from controlled experimental comparisons.

Figure 1 separates three evidence tiers: (i) descriptive historical operating-regime results, (ii) a matched comparison between RTPD-Net and constrained fine-tuning, and (iii) fold-local subject-disjoint safe-deployment evidence. This distinction is necessary because historical methods were developed under different seeds, training budgets, calibration procedures, and some different corruption settings, which are therefore used to identify operating regimes rather than to support matched statistical claims.

### 3.2. Shared Representation and Wearable-Assisted Regimes

**Temporal standardisation and radar preprocessing.** The original action segments have variable numbers of frames because participants may execute the same rehabilitation exercise at different speeds. To provide a fixed-length input while preserving the temporal ordering and relative progression of each movement, every radar and IMU segment is resampled to T=96 time steps using linear interpolation along the temporal dimension. Let an original segment contain *L* frames. The resampling operation maps the sequence from the original temporal grid {1,…,L} onto a uniformly spaced 96-step grid while leaving the spatial and feature dimensions unchanged. Thus, slower and faster executions are represented on a common normalised temporal axis, allowing the temporal encoder to compare corresponding phases of an exercise without requiring zero-padding or truncation of variable-length sequences. The choice of 96 frames provides sufficient temporal resolution for the multi-stage temporal encoder while maintaining a compact and computationally manageable sequence length across all experiments.

Importantly, the original segment length *L* (and therefore absolute movement duration) is *not* retained as an additional model feature. Consequently, the present formulation is designed primarily to recognise the spatio-temporal pattern and relative evolution of an exercise rather than to explicitly classify execution speed or duration. Temporal resampling may therefore attenuate information associated purely with absolute movement speed; incorporating original duration or execution-rate descriptors is a relevant extension for future rehabilitation-quality assessment.

**Training-only normalisation.** Normalisation parameters are estimated exclusively from the training subjects in order to prevent information leakage from validation or test participants. For radar, the mean and standard deviation are computed separately for each of the five radar feature channels after aggregating all available training-subject frames and all 14×14 spatial locations. Specifically, if $r_{n,h,w,c}$ denotes a radar value for channel c∈{1,…,5}, the channel-wise statistics are

(1) $$\mu_{c}^{r}=\frac{1}{N_{r}}\sum_{n,h,w}r_{n,h,w,c},\;\sigma_{c}^{r}=\sqrt{\frac{1}{N_{r}}\sum_{n,h,w}{\left(r_{n,h,w,c}-\mu_{c}^{r}\right)}^{2}},$$

Here, *n* indexes radar observations across the training subjects and frames, *h* and *w* index the 14×14 spatial grid locations, *c* indexes the five radar feature channels, $\mu_{c}^{r}$ and $\sigma_{c}^{r}$ denote the channel-wise mean and standard deviation, respectively, $N_{r}$ is the number of values used to estimate each channel statistic, and ϵ is a small constant added for numerical stability. The normalised radar input is then

(2) $${\hat{r}}_{n,h,w,c}=\frac{r_{n,h,w,c}-\mu_{c}^{r}}{\sigma_{c}^{r}+\epsilon },$$

When a subject recording contains more than 4000 frames, frames are sampled uniformly across that recording for estimation of the normalisation statistics, preventing disproportionately long recordings from dominating the statistics. The resulting five channel-wise means and standard deviations are fixed and subsequently applied unchanged to the training, validation, and test segments. In the grouped subject-disjoint experiments, these statistics are recomputed independently within every fold using only that fold’s training subjects.

The IMU stream is normalised analogously, but independently for each of its 72 features (six wearable sensors × 12 features); no validation- or test-subject samples contribute to these estimates.

**Radar representation learning.** After temporal resampling and normalisation, each radar frame has dimension 14×14×5. A three-stage convolutional frame encoder with batch normalisation and GELU activations extracts spatial radar features from each frame, followed by spatial pooling and linear projection to a 128-dimensional token. Applying the frame encoder independently across the 96 time steps produces a sequence

(3) $$H^{r}=\left[h_{1}^{r},\ldots ,h_{96}^{r}\right]\in R^{96\times 128}.$$

Here, $H^{r}$ is the complete radar token sequence, $h_{t}^{r}\in R^{128}$ is the radar embedding at resampled time index t∈{1,…,96}, 96 is the fixed temporal length, and 128 is the latent embedding dimension. Four residual temporal convolutional blocks with kernel size 3 and dilation factors (1,2,4,8) then progressively enlarge the temporal receptive field while preserving one representation for each time step. This dilated design allows the model to capture both local transitions and longer-range dependencies across an exercise without reducing the temporal resolution of the sequence, following the general temporal-convolutional modelling approach of Bai et al. [29]. Finally, learned temporal attention pooling assigns data-dependent importance to the 96 temporal tokens and produces the fixed-dimensional radar sequence representation used for exercise classification and subsequent knowledge distillation objectives.

**Wearable branch and multimodal teacher.** At each of the 96 time steps, the measurements from the six IMUs are concatenated to form a 72-dimensional feature vector (six sensors, ×12 features) and linearly projected into the same 128-dimensional latent space used by the radar branch. The projected IMU sequence is then subjected to *matched temporal refinement*. Here, "matched" denotes architectural correspondence with the radar temporal encoder rather than parameter sharing: the IMU branch uses its own four residual temporal convolutional blocks with kernel size 3 and dilation factors (1,2,4,8). Consequently, both modalities retain 96 temporally ordered 128-dimensional tokens,

(4) $$H^{r},H^{i}\in R^{96\times 128},$$

where $H^{r}$ and $H^{i}$ denote the refined radar and IMU token sequences, respectively. Using the same temporal resolution and embedding dimensionality makes the two streams directly compatible for subsequent cross-modal interaction while allowing each encoder to learn modality-specific temporal filters.

The multimodal teacher then applies IMU-to-radar cross-attention, using the radar tokens as queries and the temporally refined IMU tokens as the source of privileged motion information. This allows wearable-derived dynamics to condition the radar representation during training. A reliability module subsequently weights the radar and IMU evidence before producing fused teacher tokens and class logits. Importantly, the radar and IMU temporal encoders do not share weights, and the entire IMU branch is removed at deployment; the wearable stream therefore acts only as privileged training supervision, while inference remains radar-only.

**Radar self-supervision.** Before supervised distillation, the radar encoder is initialised with self-supervised learning (SSL). Two augmented radar views are generated by masking approximately 30% of temporal positions and spatial cells, applying modest amplitude jitter, and adding low Gaussian noise. The SSL objective combines masked frame-statistic reconstruction with an NT-Xent contrastive term. This stage encourages the radar branch to encode temporally stable motion structure before it is exposed to teacher supervision.

**Standard knowledge distillation.** The baseline radar-only student is trained from hard class labels together with temperature-scaled teacher logit transfer, following the conventional knowledge-distillation formulation of Hinton et al. [6],

(5) $$L_{\mathrm{KD}}=T^{2}D_{\mathrm{KL}}\;\left(\sigma \left(z_{t}/T\right)\;∥\;\sigma \left(z_{s}/T\right)\right),\;T=4.$$

Here, $z_{t}$ and $z_{s}$ denote the teacher and student class logit vectors, respectively; σ(·) is the softmax function; $D_{\mathrm{KL}}\left(p∥q\right)$ denotes the Kullback–Leibler divergence from distribution *p* to *q*; and *T* is the distillation temperature. Following the standard knowledge-distillation principle of using a temperature greater than one to soften the teacher probability distribution [6], we fixed T=4 as a moderate softening value for all KD-based experiments. Importantly, this temperature was specified globally in the experimental configuration and was not re-optimised separately for individual validation splits, subject folds, or distillation variants. The same value was therefore retained across the fixed subject split and the fold-local grouped evaluations, reducing the risk of fold-specific hyperparameter tuning and ensuring that differences between methods were attributable to their learning objectives rather than to different distillation temperatures. The student’s supervised cross-entropy loss is retained alongside the soft-target loss so that teacher supervision complements, rather than replaces, the ground-truth class labels.

**RTPD-Net.** RTPD-Net extends logit transfer to the temporal and representation levels. The multimodal teacher supplies reliability-weighted logits and fused temporal tokens; the radar student receives confidence-weighted logit KD, soft temporal alignment between student and teacher token sequences, relation matching between within-batch representations, class-prototype supervision, and a subject-adversarial classifier connected through gradient reversal. The relation-matching component is conceptually related to relational knowledge distillation, which transfers structural relationships between teacher and student representations rather than matching individual outputs alone. Its training objective is:

(6) $$L_{\mathrm{RTPD}}=L_{\mathrm{CE}}+\lambda_{l}L_{\mathrm{KD}}+\lambda_{t}L_{\mathrm{temp}}+\lambda_{r}L_{\mathrm{rel}}+\lambda_{p}L_{\mathrm{proto}}+\lambda_{a}L_{\mathrm{subj}}.$$

The individual terms are designed to transfer complementary information from the wearable-assisted multimodal teacher to the radar-only student. $L_{\mathrm{CE}}$ is the standard supervised cross-entropy loss between the student prediction and the ground-truth exercise label. $L_{\mathrm{KD}}$ is the temperature-scaled logit distillation loss defined above and transfers the teacher’s softened class-probability structure.

The temporal distillation term $L_{\mathrm{temp}}$ aligns the student radar token sequence with the temporally refined multimodal teacher sequence. Let $H^{s}={\left\{h_{t}^{s}\right\}}_{t=1}^{T}$ and $H^{t}={\left\{h_{t}^{t}\right\}}_{t=1}^{T}$ denote the student and teacher temporal representations, respectively. After $\ell_{2}$ normalisation, pairwise student–teacher temporal similarities are converted into a soft alignment distribution, and the loss penalises disagreement between corresponding temporal locations. This term therefore encourages the radar-only student to preserve exercise-phase information conveyed by the wearable-assisted teacher rather than matching only the final class logits.

The relation term $L_{\mathrm{rel}}$ transfers the geometry of the representation space. Let $u_{i}^{s}$ and $u_{i}^{t}$ denote the pooled student and teacher embeddings, respectively, for sample *i* in a mini-batch of size *B*. The embeddings are first $\ell_{2}$-normalised,

(7) $${\hat{u}}_{i}^{s}=\frac{u_{i}^{s}}{∥u_{i}^{s}{∥}_{2}},\;{\hat{u}}_{i}^{t}=\frac{u_{i}^{t}}{∥u_{i}^{t}{∥}_{2}}.$$

Pairwise Euclidean-distance matrices are then constructed as

(8) $$D_{ij}^{s}={∥{\hat{u}}_{i}^{s}-{\hat{u}}_{j}^{s}∥}_{2},\;D_{ij}^{t}={∥{\hat{u}}_{i}^{t}-{\hat{u}}_{j}^{t}∥}_{2},\;i,j=1,\ldots ,B.$$

Their batch-wise mean distances are

(9) $${\bar{D}}^{s}=\frac{1}{B^{2}}\sum_{i=1}^{B}\sum_{j=1}^{B}D_{ij}^{s},\;{\bar{D}}^{t}=\frac{1}{B^{2}}\sum_{i=1}^{B}\sum_{j=1}^{B}D_{ij}^{t},$$

where the full B×B matrices, including their zero-valued diagonal entries, are included in the mean, consistent with the implementation. The scale-normalised relation matrices are therefore

(10) $${\tilde{D}}^{s}=\frac{D^{s}}{\max({\bar{D}}^{s},\epsilon )},\;{\tilde{D}}^{t}=\frac{D^{t}}{\max({\bar{D}}^{t},\epsilon )},\;\epsilon =10^{-6}.$$

The relation-distillation loss is then the element-wise Smooth-L1 discrepancy between the normalised student and teacher relation matrices,

(11) $$L_{\mathrm{rel}}=\frac{1}{B^{2}}\sum_{i=1}^{B}\sum_{j=1}^{B}\ell_{\mathrm{SL}1}\left({\tilde{D}}_{ij}^{s}-{\tilde{D}}_{ij}^{t}\right),$$

where

(12) $$\ell_{\mathrm{SL}1}\left(e\right)=\left\{\begin{array}{cc} \frac{1}{2}e^{2}, & |e|<1,\\ \left|e\right|-\frac{1}{2}, & |e|\geq 1. \end{array}\right.$$

Thus, small discrepancies between student and teacher pairwise relations are penalised quadratically, whereas larger discrepancies receive a linear penalty, making the relation-matching objective less sensitive to large individual errors than a purely squared loss. The teacher relation matrix is treated as a detached target, so gradients are propagated only through the student representations. This component is conceptually related to relational knowledge distillation because it transfers structural relationships between samples rather than matching individual representations directly.

The prototype term $L_{\mathrm{proto}}$ encourages each radar embedding to remain close to the corresponding teacher-derived class prototype. A prototype $p_{c}$ is obtained by averaging the pooled teacher representations belonging to class *c* in the training set and subsequently normalising the resulting vector. For a student embedding $u_{i}^{s}$, prototype logits are obtained from cosine similarities,

(13) $$q_{ic}=\frac{\langle {\hat{u}}_{i}^{s},{\hat{p}}_{c}\rangle }{\tau_{p}},\;L_{\mathrm{proto}}=-\frac{1}{B}\sum_{i=1}^{B}\log\frac{\exp(q_{iy_{i}})}{\sum_{c=1}^{C}\exp\left(q_{ic}\right)},$$

Here, i∈{1,…,B} indexes samples in the mini-batch, c∈{1,…,C} indexes exercise classes, *B* is the mini-batch size, *C* is the number of classes, $u_{i}^{s}$ is the pooled student embedding for sample *i*, $p_{c}$ is the teacher-derived prototype for class *c*, ${\hat{u}}_{i}^{s}$ and ${\hat{p}}_{c}$ denote their $\ell_{2}$-normalised forms, $q_{ic}$ is the temperature-scaled cosine-similarity logit between sample *i* and class prototype *c*, and $y_{i}$ is the ground-truth class label of sample *i*. This provides class-level semantic guidance in addition to instance-level logit and temporal supervision.

Finally, $L_{\mathrm{subj}}$ is an adversarial subject-classification loss. A gradient-reversal layer connects the student’s pooled radar representation to an auxiliary classifier that predicts the training-subject identity. The auxiliary classifier itself is trained to recognise the subject, while the reversed gradient encourages the radar encoder to suppress subject-specific information. The objective is therefore to promote exercise-discriminative but subject-invariant representations, which is particularly relevant to cross-subject generalisation.

The loss coefficients were fixed as

(14) $$\left(\lambda_{l},\lambda_{t},\lambda_{r},\lambda_{p},\lambda_{a}\right)=\left(0.90,\;0.45,\;0.20,\;0.45,\;0.08\right).$$

These coefficients were established during method development to keep the supervised and logit-distillation objectives dominant while allowing the temporal and prototype terms to contribute meaningful auxiliary supervision, assigning a smaller weight to relation matching and a deliberately weak weight to the adversarial subject objective to avoid destabilising class discrimination. They were fixed before final test evaluation and were not independently re-optimised for individual test subjects, corruption conditions, or grouped subject folds. The same weighting scheme was therefore used throughout the RTPD-Net experiments, so differences between evaluation settings cannot be attributed to fold-specific tuning of the loss coefficients. Importantly, we did not conduct an exhaustive hyperparameter search over these weights; the later equal-budget ablation showed that the complete structural objective did not dominate standard logit KD under every setting. This observation directly motivated the final constrained fine-tuning experiment, in which robustness was improved through a targeted adaptation stage rather than by adding further simultaneous loss terms.

**Complementary operating regimes.** PARETO-RKD introduces counterfactual reliability supervision by presenting the multimodal teacher with clean, radar-corrupted, and IMU-corrupted views. Radar degradation is modelled using temporal frame masking, contiguous temporal-block removal, spatial masking, radar-channel removal, additive Gaussian noise, amplitude attenuation, and temporal displacement/rolling, representing packet loss, temporary signal interruption, reduced spatial visibility, receiver-channel failure, measurement interference, weak radar returns, and timing disturbances, respectively. The corresponding IMU reliability tests perturb the wearable stream using additive Gaussian noise and partial or complete sensor/modality removal, thereby simulating noisy inertial measurements and unavailable wearable information. PARETO-RKD additionally trains the radar student against difficult or worst-performing corrupted radar views, applies consistency and robust knowledge-distillation objectives, routes structural auxiliary losses only when their gradients agree with the primary objective, and retains standard KD as a validation-only safety anchor.

AURORA-RKD evaluates related degradation conditions through radar AugMix and worst-view robustness training and explicitly stress-tests the multimodal teacher under gradual IMU noise and complete modality loss. In particular, additive Gaussian perturbation is used to examine progressive degradation of the IMU stream, while complete radar or IMU removal tests whether the quality-aware fusion mechanism correctly transfers its weight to the surviving modality. For radar-only robustness, the evaluated perturbations include frame loss/masking, spatial masking, radar-channel removal, additive Gaussian noise, amplitude attenuation, and temporal perturbation. The AURORA framework further incorporates multi-kernel and self-attention radar branches, quality-aware multimodal fusion, hard missing-modality overrides, candidate diversity, non-negative aggregation, and post hoc uncertainty analysis. Its multi-kernel temporal feature extraction is related in general design principle to multiscale time-series classifiers such as InceptionTime [30]; this citation does not imply that the AURORA architecture is identical to InceptionTime.

Because the historical PARETO-RKD and AURORA-RKD experiments used slightly different corruption suites, severities, and training procedures, their corruption results are interpreted as complementary operating-regime evidence rather than as a directly matched robustness comparison. PARETO-RKD primarily characterises corruption-retention and cross-subject robustness, whereas AURORA-RKD characterises calibration, gradual sensor-quality degradation, and complete missing-modality behaviour. Exact corruption definitions, severity levels, subject splits, and optimisation hyperparameters are provided in the Supplementary Material.

### 3.3. Constrained RTPD Fine-Tuning

The final experiment introduces no new architecture. Let $\theta_{0}$ denotes the trained RTPD radar-only student and θ a fine-tuned copy initialised from $\theta_{0}$. For each clean radar sequence *x* with an exercise label *y*, two independently corrupted views, ${\tilde{x}}_{1}$ and ${\tilde{x}}_{2}$, are generated online during fine-tuning.

**Corruption sampling policy.** Each corrupted view is produced by applying one operator selected independently and uniformly at random from the seven-member corruption set

$$\begin{array}{cc} C=\{ & \mathrm{frame}\;\mathrm{masking},\mathrm{temporal}\text{-}\mathrm{block}\;\mathrm{masking},\mathrm{spatial}\;\mathrm{masking},\mathrm{channel}\;\mathrm{removal},\\ \; & \mathrm{gaussian}\;\mathrm{noise},\mathrm{amplitude}\;\mathrm{attenuation},\mathrm{temporal}\;\mathrm{rolling}\}. \end{array}$$

The operator is sampled separately for every sample in a mini-batch and separately for ${\tilde{x}}_{1}$ and ${\tilde{x}}_{2}$. Consequently, the two views of the same clean sequence may contain either different corruption types or, by chance, the same type with independently sampled corruption locations or noise realisations. Only one corruption operator is applied to a given corrupted view; the training views are therefore not formed by sequentially composing multiple corruption types.

Corruption strength follows a deterministic curriculum across the fine-tuning epochs. For epoch e∈{1,…,E}, the severity is

(15) $$s_{e}=s_{\min}+\frac{e-1}{E-1}\left(s_{\max}-s_{\min}\right),$$

where $s_{\min}=0.08$ and E=10. The maximum curriculum severity depends on the fine-tuning recipe: $s_{\max}=0.30$ for preserve_clean, $s_{\max}=0.40$ for balanced_robust, and $s_{\max}=0.45$ for strong_robust. Thus, corruption intensity increases gradually during optimisation instead of exposing the model immediately to the strongest perturbations. Within a given epoch, both corrupted views use the same curriculum severity $s_{e}$, while their corruption type, affected frames or spatial locations, removed channels, noise samples, or temporal shifts are generated independently.

The severity parameter has an operator-specific interpretation. For frame masking and contiguous temporal masking, it determines the fraction of the 96 time steps removed. For spatial masking, it determines the area of the spatial region suppressed; for channel removal, it controls the proportion of the five radar channels removed. For Gaussian corruption, the noise standard deviation increases with severity; amplitude corruption progressively attenuates the radar magnitude; and temporal rolling increases the allowable temporal displacement. This common severity variable therefore provides a consistent curriculum parameter while allowing each corruption to retain its physically appropriate implementation. Table 1 gives the exact mapping used by the implementation, where s∈[0,1] is a dimensionless corruption-severity parameter (not a unit of time), T=96 frames, and the radar tensor has H=W=14 and C=5 channels.

**Figure 2:**
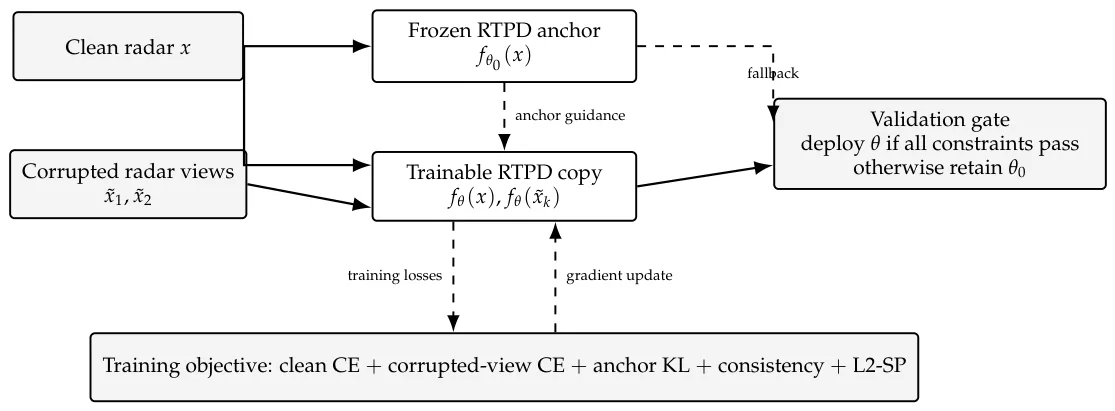
Constrained fine-tuning of the unchanged RTPD radar-only student. Clean radar is processed by both the frozen anchor and trainable copy, whereas corrupted radar views are presented only to the trainable copy. The frozen anchor provides reference predictions for the anchoring objectives but receives no gradient update. The trainable RTPD copy contributes clean and corrupted predictions to the training objective, which in turn updates only the trainable copy. Deployment uses the adapted checkpoint only when all predefined validation constraints are satisfied; otherwise, the original RTPD model is retained.

The frozen anchor preserves the original clean decision function, whereas the trainable copy is exposed to the clean sequence and both corrupted views. Figure 2 shows this training and validation logic.

Define the softened frozen-anchor and trainable-model distributions as

(16) $$p_{0}\left(x;T_{a}\right)=\sigma \;\left(\frac{f_{\theta_{0}}\left(x\right)}{T_{a}}\right),\;p_{\theta }\left(x;T_{a}\right)=\sigma \;\left(\frac{f_{\theta }\left(x\right)}{T_{a}}\right),$$

where $T_{a}=3$ is the anchor-distillation temperature, used only during constrained fine-tuning. For the clean, ‘corrupted consistency’ term, we instead use the native predictive distributions

(17) $$q_{\theta }\left(x;T_{c}\right)=\sigma \;\left(\frac{f_{\theta }\left(x\right)}{T_{c}}\right),\;T_{c}=1,$$

and denote them by sg[·] the stop-gradient operator. The clean prediction therefore acts as a fixed target within the consistency term,

(18) $${\bar{q}}_{\theta }\left(x\right)=\mathrm{sg}\;\left[q_{\theta }\left(x;1\right)\right].$$

The first two terms are supervised exercise-label cross-entropies for the clean and corrupted student predictions, respectively. The third term preserves the softened, clean decision distribution of the frozen RTPD anchor, while the fourth transfers the same anchor distribution to the corrupted student views. The fifth term is a clean-corrupted prediction-consistency loss: the clean prediction is detached and used as the target distribution, so this term drives the corrupted prediction towards the current clean prediction without back-propagating through the clean target. The final L2-SP term constrains parameter drift from the trusted RTPD solution. The constrained fine-tuning objective is

(19) $$\begin{array}{cc} L_{\mathrm{CFT}}= & \alpha \;L_{\mathrm{CE}}\;\left(f_{\theta }\left(x\right),y\right)+\frac{\beta }{2}\sum_{k=1}^{2}L_{\mathrm{CE}}\;\left(f_{\theta }\left({\tilde{x}}_{k}\right),y\right)\\ \; & +\gamma T_{a}^{2}D_{\mathrm{KL}}\;\left(p_{0}\left(x;T_{a}\right)\;∥\;p_{\theta }\left(x;T_{a}\right)\right)\\ \; & +\frac{\delta T_{a}^{2}}{2}\sum_{k=1}^{2}D_{\mathrm{KL}}\;\left(p_{0}\left(x;T_{a}\right)\;∥\;p_{\theta }\left({\tilde{x}}_{k};T_{a}\right)\right)\\ \; & +\frac{\eta }{2}\sum_{k=1}^{2}D_{\mathrm{KL}}\;\left({\bar{q}}_{\theta }\left(x\right)\;∥\;q_{\theta }\left({\tilde{x}}_{k};1\right)\right)+\lambda {∥\theta -\theta_{0}∥}_{2}^{2}. \end{array}$$

The use of $T_{a}=3$ in constrained fine-tuning is distinct from the standard RTPD knowledge-distillation temperature $T_{\mathrm{KD}}=4$ used in (Equation (5)). These temperatures correspond to different training stages and serve different purposes: $T_{\mathrm{KD}}=4$ softens the multimodal teacher logits during initial training of the radar-only RTPD student, whereas $T_{a}=3$ provides a moderately softened reference distribution from the already trained, frozen RTPD model during robustness fine-tuning. We fixed both values as stage-specific hyperparameters and kept them unchanged across the corresponding experiments and subject folds; we make no claim that they are globally optimal temperature values.

The consistency term intentionally uses $T_{c}=1$ rather than $T_{a}=3$. Unlike the anchor KL terms, which aim to preserve inter-class information contained in softened anchor probabilities, the consistency term is designed to enforce agreement between the model’s actual clean and corrupted predictive distributions at the native inference scale. Temperature softening is therefore not applied to this term. This also matches the implementation, in which ordinary softmax probabilities are computed from the clean and corrupted logits without division by a temperature.

The validation-selected objective uses

$$\left(\alpha ,\beta ,\gamma ,\delta ,\eta ,\lambda \right)=\left(0.40,0.45,0.85,0.35,0.18,2\times 10^{-5}\right),$$

with $T_{a}=3$, a learning rate of $4\times 10^{-5}$, and 10 fine-tuning epochs. A checkpoint is accepted only when validation macro-F1 is no more than 0.005 below the RTPD anchor, validation accuracy is no more than 0.01 below the anchor, ECE increases by at most 0.03, and mean corrupted macro-F1 improves. Failure of any condition triggers deployment of the unchanged anchor model $\theta_{0}$.

## 4. Experimental Setup

### 4.1. Dataset, Splits, and Clinical Scope

We use the P2_10 subset of mRI: 20 subjects, 10 exercise classes, and one 96-frame segment per class and subject (200 segments). Radar tensors are 96×14×14×5; six IMUs with 12 features each, flattened to 96×72. The fixed split uses 12 training subjects (03, 04, 05, 06, 08, 09, 11, 12, 13, 17, 18, 20), four validation subjects (02, 07, 14, 16), and four test subjects (01, 10, 15, 19), yielding 40 balanced test segments. The grouped protocol contains five subject-disjoint folds, each with 12 training, four validation, and four test subjects; normalisation, SSL, teacher, student, calibration, and fine-tuning are recomputed inside each fold. Because participants are healthy and each class has one segment per subject, the study evaluates algorithmic exercise recognition, not patient outcomes or exercise-quality assessment.

**Acquisition devices and wearing protocol.** We analyse the released mRI recordings and did not collect new sensor data. In the original mRI acquisition, a Texas Instruments IWR1443 Boost mmWave radar recorded point clouds at 10 Hz, while six Wit-motion BWT901CL IMUs sampled inertial data at 50 Hz [1]. The radar and RGB-D sensors were positioned on a desk approximately 2.4 m from the participant. The six IMUs were tightly attached to the left wrist, right wrist, left knee, right knee, head, and pelvis. Each unit contains a three-axis accelerometer, a gyroscope, and a magnetometer; the released representation used here provides orientation and acceleration features. These acquisition details clarify the physical origin and placement of the privileged wearable stream; our models operate only on the released, synchronised mRI features.

### 4.2. Optimisation, Corruptions, and Evaluation

Models use AdamW with batch size 24, initial learning rate $3\times 10^{-4}$ for the main RTPD stages, weight decay $10^{-4}$, label smoothing 0.03, mixed-precision training, and global gradient L2-norm clipping at 5.0 on an NVIDIA Tesla P100 GPU. RTPD uses 10 SSL epochs, 20 teacher epochs, and 22 student epochs with validation-based early stopping; constrained fine-tuning uses 10 epochs at $4\times 10^{-5}$. Reported metrics are accuracy, balanced accuracy, macro-F1, ECE, negative log-likelihood, and multiclass Brier score. ECE is computed from confidence bins as the sample-frequency-weighted absolute gap between bin accuracy and mean confidence.

The corruption suite comprises frame loss, contiguous temporal blocks, spatial masking, channel dropping, Gaussian noise, amplitude attenuation, and temporal rolls. The original single-reconstruction fixed-split experiment assessed its clean macro-F1 difference using a paired 40-segment bootstrap (2000 draws) and segment-wise permutation (5000 draws); those exploratory segment-level intervals should not be interpreted as independent-subject uncertainty. The subject-level inferential procedure for corrupted macro-F1 is specified separately below. All original fixed-split results derive from one model initialisation per configuration; historical PARETO/AURORA protocols differ. The preceding single-partition five-fold evaluation is also retained for transparency, while a wholly repeated five-partition-seed evaluation is reported separately in Section 5.5. 

**Seeded checkpoint re-evaluation.** The saved RTPD student and CFT weights were loaded with strict architecture checks. Both received exactly matched corrupted tensors for each of 11 held-out radar-degradation conditions, repeated with five fixed-seed realisations. The evaluation archives 40×11×5=2200 paired corrupted predictions in addition to 40 clean predictions. Mean corrupted macro-F1 is the unweighted mean of the per-condition, per-realisation class-macro-F1 scores. A paired subject-cluster bootstrap resamples whole subjects (all ten class labels and all corruption realisations together; 5000 draws). A complementary exact paired model-swap test exchanges the two sets of predictions jointly for each whole subject; with four subjects there are only $2^{4}=16$ swap patterns. Consequently, the minimum attainable two-sided exact *p*-value is 0.125 for a positive effect on all four subjects. The bootstrap interval should be treated as descriptive with only four independent clusters, not as stronger evidence than the exact test. The saved CFT checkpoint and independently seeded corruption realisations yield different numbers from the previous single-realisation headline evaluation, so they are presented as a separate re-evaluation rather than substituted without explanation. 

**Repeated full subject-partition evaluation.** Five distinct partition seeds (101, 202, 303, 404, 505) each define five subject-disjoint folds with 12 training, 4 validation, and 4 test subjects. Every seed tests each of the 20 subjects exactly once. Within each of the 25 fold-local pipelines, training-only normalisation, radar SSL, multimodal teacher training, RTPD student training, calibration, and constrained fine-tuning are rerun. Checkpoints are selected solely on the fold validation subjects, with the unchanged RTPD anchor substituted if the fine-tuned candidate fails the gate. Each fold is evaluated with its own test loader, 11 degradation conditions, and three fixed corruption realisations. Paired differences are first aggregated over the five folds within each partition seed; descriptive bootstrap intervals then resample these five seed-level summaries, rather than incorrectly treating the 25 overlapping folds as independent test cohorts. Because all seeds reuse the same 20 people, these intervals measure sensitivity to partitioning and training randomness conditional on this cohort, not uncertainty for a new population of patients. 

## 5. Results and Discussion

### 5.1. Cross-Experiment Operating Regimes

Table 2 summarises operating regimes rather than a single matched statistical ranking. The upper block contains historical results produced under different development protocols; it is used only to identify characteristic strengths. The lower block is the controlled final comparison, in which RTPD-Net and constrained fine-tuning share the same split, corruption suite, calibration procedure, and evaluation code.

Standard KD raised radar-only macro-F1 from 0.4230 to 0.6530, providing descriptive evidence that IMU supervision can transfer useful motion information. RTPD-Net then improved clean recognition through temporal and representation-level transfer; however, its equal-budget ablation showed standard logit KD (0.7825) outperforming the complete structural objective (0.7394). Within its own historical protocol, PARETO-RKD showed stronger corruption retention than its clean-oriented counterparts; however, its selector chose the standard-KD anchor (α=0), preventing a weaker clean-validation solution from being deployed. Within its own historical protocol, AURORA-RKD yielded ECE 0.0889 and handled complete missing modalities through hard overrides. Because these historical runs are unmatched, these observations characterise method behaviour and are not used to infer superiority between methods.

#### Within-Method Variation and Effect-Size Context

The previously reported RTPD clean macro-F1 changes from 0.8569 in its historical run to 0.7902 in the final matched reference (absolute difference 0.0667). Standard KD changes from 0.6530 in its historical experiment to 0.7825 in the equal-budget component ablation (0.1295) and hard-label radar training from 0.4230 to 0.5783 (0.1553). These are between-protocol observations, not estimates of a controlled random-seed standard deviation: the budgets, selection procedures, and sometimes stochastic data/initialisation differ. Nonetheless, their 0.0667–0.1553 range is 3.3–7.6 times the original matched clean gain of 0.0204; such sensitivity makes a small single-run clean gain unsuitable as evidence of generalisable superiority. A proper within-protocol run-to-run variance would require independently repeated model initialisations on identical folds. The five partition seeds below instead quantify variation across subject partitions and training runs conditional on the same cohort.

### 5.2. Matched Constrained Fine-Tuning

Constrained RTPD-FT improved clean accuracy by 2.5 percentage points and macro-F1 by 0.0204. More importantly for the deployment objective, the mean corrupted macro-F1 increased by 0.1613 and the worst-case macro-F1 by 0.1847. Temporal-block macro-F1 increased from 0.2737 to 0.7998, 50% frame masking from 0.3016 to 0.4879, 40% channel removal from 0.3156 to 0.4584, and 50% amplitude attenuation from 0.3327 to 0.5945. ECE improved from 0.1568 to 0.1263, while 40% channel removal remained the weakest evaluated condition. For the clean macro-F1 difference, the observed Δ=0.0204 had a 95% paired-bootstrap percentile interval of [−0.0812,0.1180] and a two-sided paired permutation p=0.743. The results therefore show an observed multi-objective shift on this fixed split. However, the clean-performance difference was not statistically significant. For this historical single-realisation corruption estimate, no subject-level inferential test was performed; the separate seeded checkpoint evaluation below supplies subject-level uncertainty for the five-realisation corruption estimate.

**Figure 3:**
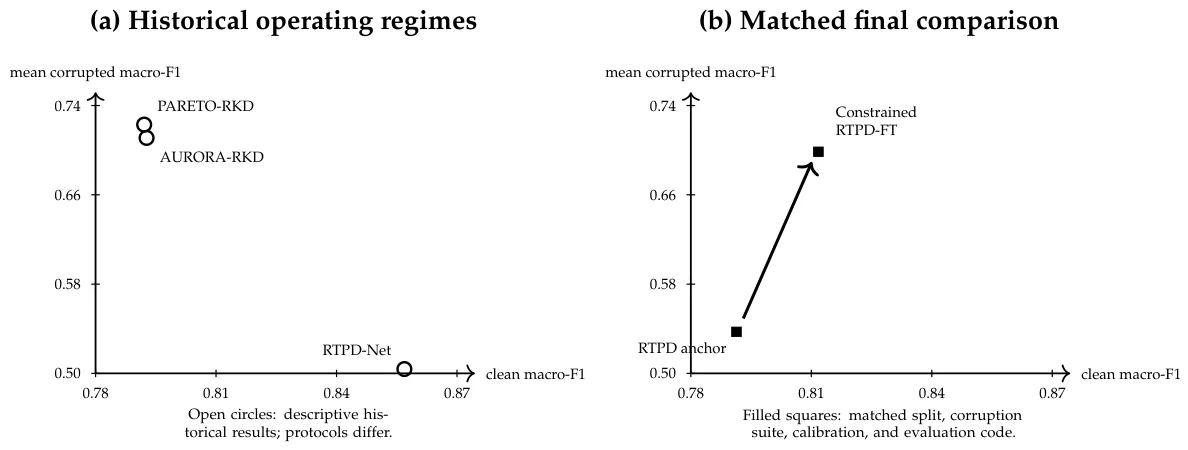
Multi-objective operating-regime visualisation. (**a**) Descriptive historical results obtained under different optimisation and corruption protocols; open markers should not be interpreted as a matched statistical ranking. (**b**) The matched comparison uses the same split, corruption suite, calibration protocol, and evaluation code. Both panels use identical axis ranges (clean macro-F1: 0.78–0.87; mean corrupted macro-F1: 0.50–0.74) to avoid visual distortion between historical and matched results. The straight arrow in panel (**b**) indicates the observed shift from the RTPD anchor to constrained RTPD-FT.

**Paired corruption-robustness inference.** The fixed-split checkpoint re-evaluation using five corruption seeds per condition gives a reference mean corrupted macro-F1 of 0.5370 and a CFT mean of 0.6374 (paired difference of +0.1004). The subject-cluster bootstrap percentile interval is [0.0683, 0.1283] from 5000 resamples, while the exact two-sided subject-level model-swap test gives p=0.125 from 16 possible assignments. The bootstrap interval is conditional on the four observed subjects and does not establish a population-level improvement: with only four test subjects, cluster resampling cannot adequately represent wider population variation. The clean re-evaluation changes accuracy from 0.800 to 0.825, precisely one additional correctly recognised segment, and clean macro-F1 from 0.7902 to 0.8150. Relative to the original reported mean corrupted 0.5332 to 0.6945 (+0.1613), the re-evaluation yields a smaller +0.1004 gain: different stochastic corruption realisations and the checkpoint used in this evaluation prevent treating the two numbers as a replicated identical estimate. The improvement is concentrated in some perturbations (notably temporal-block masking); Gaussian noise at severity 0.25 instead changes macro-F1 by −0.0232. Table 3 summarises the replicated evaluation, and all conditions and realisations are reported in Supplementary File S1. Figure 3 distinguishes the descriptive historical operating regimes from the matched fixed-split comparison; the two panels use identical axis ranges. 

#### Checkpoint and Evaluation Provenance

Table 4 reconciles the three corruption summaries. They are different evaluation objects, not repeated measurements of one checkpoint under an unchanged protocol. The 0.5332/0.6945 pair is the original matched fixed-split RTPD/strong-robust-CFT evaluation using one set of stochastic corruption draws. The 0.5452/0.6731 pair belongs to the separate objective-ablation block, where the CFT row is the *anchor + robust + consistency* candidate and the corruption realisation differs. The 0.5370/0.6374 pair is a subsequent independent five-realisation-per-condition evaluation: the input state dictionaries were loaded from rtpdnet_radar_student.pt and checkpoint_strong_robust.pt, respectively. The checkpoint file names document which state dictionaries were read, but a raw state dictionary lacks training metadata sufficient to prove that the CFT weights correspond to the original reported *strong_robust* selection or the ablation candidate. We therefore do not equate these checkpoint identities or use their scores as interchangeable replication estimates. The clean macro-F1 of 0.8106 is the original matched CFT evaluation, whereas 0.8150 occurs in the ablation/loaded-checkpoint evaluations, under their own evaluation paths.

### 5.3. Objective Ablation and Validation-Safe Selection

The objective ablation tests whether robustness gains arise from corrupted-view supervision alone or from the full clean-preservation constraint. Each trainable variant uses the same RTPD architecture and differs only in the fine-tuning objective. Acceptance is determined exclusively from the predefined validation constraints on clean macro-F1, clean accuracy, ECE, and mean corrupted macro-F1; the test set is evaluated only after each checkpoint has been fixed and is never used to choose the objective. The non-adapted RTPD row is therefore labelled *Reference*, not *Accepted*: it is not subjected to the acceptance gate and serves as the fallback deployment model whenever a fine-tuned candidate is rejected.

Selection proceeds in two stages. First, a checkpoint is eligible only if it satisfies all four validation constraints: clean macro-F1 may decrease by no more than 0.005 relative to the RTPD anchor, clean accuracy may decrease by no more than 0.01, ECE may increase by no more than 0.03, and mean corrupted macro-F1 must improve over the anchor. Among the validation-compliant checkpoints, the selected checkpoint is the one with the highest validation-only composite score,

(20) $$S_{\mathrm{val}}=0.45F_{1,\mathrm{clean}}+0.45F_{1,\mathrm{corr}}^{\mathrm{mean}}+0.10F_{1,\mathrm{corr}}^{\mathrm{worst}}-0.05\;\mathrm{ECE}+0.10\max\left(0,\Delta F_{1,\mathrm{corr}}^{\mathrm{mean}}\right),$$

where $F_{1,\mathrm{clean}}$ is the validation clean macro-F1, $F_{1,\mathrm{corr}}^{\mathrm{mean}}$ and $F_{1,\mathrm{corr}}^{\mathrm{worst}}$ are the mean and worst validation corrupted macro-F1 values, respectively, and $\Delta F_{1,\mathrm{corr}}^{\mathrm{mean}}$ denotes the gain in mean corrupted macro-F1 over the corresponding RTPD anchor. The same rule is first used to identify the best validation-compliant epoch within each candidate and then to select among multiple accepted fine-tuning candidates. If no candidate satisfies the gate, the system retains the original RTPD checkpoint. Thus, neither candidate selection nor checkpoint selection uses test-set performance.

**Gate-sensitivity scope.** The clean macro-F1 threshold can be partially audited from the archived validation summaries: the selected anchor+robust+consistency variant is only 0.0019 below the RTPD validation anchor (0.7667 versus 0.7686), so it remains eligible for clean-macro-F1 tolerances of 0.002, 0.005, or 0.010. By contrast, the anchor+robust-CE and strict-clean-preserving variants are more than 0.025 below the anchor and would still fail this criterion at a 0.010 tolerance. However, the archived fold summaries do not retain all per-epoch validation accuracy and ECE values required to recompute the complete four-criterion gate under alternative threshold combinations. We therefore do not claim that the observed 2/5 fold acceptance rate is invariant to the design-selected thresholds or selection-score weights; it should be interpreted as conditional on the prespecified gate used here. A full multi-threshold and multi-partition sensitivity study is identified as future validation rather than reconstructed post hoc from incomplete summaries.

Table 5 illustrates the importance of this rule. Robust-CE-only produced the strongest test row, but it failed at least one predefined validation criterion and was rejected. The validation-compliant anchor-robust-consistency objective was selected for grouped evaluation because it satisfied all constraints while providing a substantial validation robustness gain. The small numerical difference between the RTPD corruption summary in Table 2 and Table 5 arises because the ablation block uses its own deterministic stochastic-corruption realisation; objective selection is based only on its corresponding validation results. Clean preservation is ‘Accepted’ even though, on the test set, it performs worse than the RTPD reference in ECE and mean corrupted macro-F1 and has the worst corrupted macro-F1. Robust CE only is ‘Rejected’ even though it gives the best test result in every displayed metric. Anchor + robust + consistency is selected even though Robust CE only has better displayed test macro-F1, ECE, mean corrupted macro-F1, and worst corrupted macro-F1.

### 5.4. Earlier Single-Partition Subject-Disjoint Evaluation

This subsection retains the original one-seed fold-local evaluation for transparency; the five-seed validation is reported separately in Section 5.5. The numerical results from this earlier protocol should not be mixed with the five-seed protocol in a single pooled average. 

Each grouped fold independently recomputed training-subject normalisation and retrained Radar SSL, the multimodal teacher, RTPD-Net, temperature scaling, and constrained fine-tuning. The fold-local RTPD-Net row in Table 6 is the reference policy. The adapted checkpoint was approved by the predefined validation gate in two of five folds; in the remaining three folds, the safe policy automatically deployed the unchanged fold-local RTPD reference. Thus, the fine-tuning acceptance rate is 40% (2/5 folds), not a percentage of test samples.

Across the five subject-disjoint folds, the safe policy produced 0.780±0.065 accuracy and 0.7712±0.0685 macro-F1, compared with 0.765±0.091 and 0.7564±0.0896 for fold-local RTPD-Net, These averages should not be read as a stable fold-wise improvement: Fold 1 accounts for most of the clean macro-F1 increase (0.6277 to 0.6973), Folds 2–4 deploy the unchanged RTPD anchor, and Fold 5 changes only from 0.8433 to 0.8479. Mean corrupted macro-F1 changes from 0.5602±0.0748 to 0.5728±0.0715, while ECE remains similar. Because threefold use the anchor is unchanged and the experiment uses only one five-fold subject partition seed, the result is evidence that the validation gate can avoid forcing an unaccepted update, not evidence of a consistent cross-subject fine-tuning benefit.

Taken together, the experiments provide qualified answers to the research questions. Wearable supervision is associated with improved radar-only recognition in the evaluated settings (RQ1), while the historical variants illustrate different deployment regimes rather than a matched ranking (RQ2). For RQ3, constrained fine-tuning yields an observed matched robustness improvement and can be deployed selectively when validation permits; however, only two of five subject partitions approved the adapted model and the grouped experiment uses a single partition seed. The evidence therefore supports validation-gated deployment as a safety mechanism, while remaining insufficient to claim a consistent fine-tuning benefit across arbitrary unseen-subject partitions.

### 5.5. Repeated Five-Seed Subject-Disjoint Evaluation

The repeated evaluation comprises all 25 completed fold-local experiments (five folds for each of five partition seeds). Fine-tuning passed the validation gate in only 5/25 folds (20%); the remaining 20 deployments used the unmodified RTPD reference. Averaging the five within-seed means gives a clean macro-F1 of 0.7189 for the reference and 0.7218 for the gated policy (+0.0029), and mean corrupted macro-F1 of 0.5375 versus 0.5449 (+0.0074). A five-seed resampling interval for the mean corrupted difference is [0.0003, 0.0170] and for clean macro-F1 [-0.0010, 0.0076]. These intervals are conditional on the same 20 participants and should not be interpreted as a subject-generalisation significance test. Table 7 reports the seed-wise results; the complete fold-wise data and evaluation specifications are in Supplementary File S1. 

Compared with the earlier single-partition five-fold policy gain of +0.0126 mean corrupted macro-F1, the repeated-seed gain is +0.0074; both are much smaller than the fixed-split checkpoint re-evaluation (+0.1004) and the earlier single-realisation fixed-split increase (+0.1613). The discrepancy is consistent with sensitivity to the identity of held-out subjects, training randomness, corruption draws, calibration, and validation-gated fallback, rather than evidence of uniform robustness improvements. One seed (202) also shows a slight mean clean-F1 decrease despite positive mean corrupted gain; this is permissible because the gate tests the validation fold and cannot guarantee improved test outcomes. Accordingly, the main supported conclusion is selective adaptation, not a consistent cross-subject improvement. 

Table 8 reports the paired clean and corrupted macro-F1 differences for all 25 folds, distinguishing the five accepted updates from the 20 anchor fallbacks.

The 25 rows in Table 8 include five accepted fine-tunings and 20 fallbacks; any zero for a fallback is a consequence of identical model predictions rather than evidence that a fine-tuned model performed equally well. Accepted folds exhibit heterogeneous improvements, including at least one negative clean delta. The average across all folds is attenuated by fallback in 80% of folds. Because each of the five partition seeds reuses the same 20 people, the fold records are not 25 independent cohorts; seed-level summaries and conditional intervals appear in Table 7.

## 6. Conclusions

This study demonstrates a practical wearable-assisted training formulation in which the IMU contributes privileged information during training but radar remains the only sensor used at inference. The repeated evaluations show a largely non-degrading fallback policy, not demonstrating the practical utility of the gate or the superiority of an individual network or a universal benefit of constrained fine-tuning. The historical RTPD, PARETO, and AURORA numbers originate from unequal experimental protocols; they illustrate possibilities for clean recognition, corruption resistance, and calibration but cannot answer which method is best under a matched benchmark (RQ2 is descriptive). 

For the fixed 40-segment test split, a seeded five-realisation matched corruption evaluation estimated mean corrupted macro-F1 at 0.5370 for RTPD and 0.6374 for a saved CFT checkpoint, an observed difference of +0.1004. The subject-cluster bootstrap interval was conditional on the observed four subjects; the exact two-sided subject-level permutation test was not significant (p=0.125), reflecting the fundamental limitation of four held-out subjects. Across five partition seeds and 25 fully retrained grouped folds, the mean corrupted macro-F1 improvement of the validation-gated deployment policy was only +0.0074, with fine-tuning accepted in 5 folds and rejected in 20. These findings weaken the earlier impression of a stable cross-subject gain. The supported policy conclusion is limited: fallback makes rejected adaptations harmless relative to the anchor, but the 5/25 acceptance frequency and small mean gain do not establish that the gate is beneficial; superiority across individuals, sensors, and clinical populations remains to be established. 

## 7. Limitations and Future Work

The fixed split includes only four independent test subjects, one segment per class per subject; repeated corruption draws create measurement realisations, not additional independent subjects. Consequently, the 95% subject-cluster bootstrap interval may be overly optimistic, and the exact two-sided subject-swap test has only 16 possible assignments. The repeated-five-seed analysis expands assessment of partition sensitivity, but the seeds reuse the same 20 participants; its seed-bootstrap interval must not be interpreted as a population-level confidence interval. In addition, the saved CFT checkpoint and independently generated corruption draws do not reproduce the original 0.6945 mean corrupted score. The original single-realisation comparisons and the seeded checkpoint re-evaluation are therefore presented separately, with the lower +0.1004 gain reported explicitly. The grouped policy accepted fine-tuning only 20% of the time, and improvements on one fixed test split did not transfer in magnitude to the grouped evaluation. 

The historical RTPD-Net, PARETO-RKD, and AURORA-RKD methods were not jointly retrained under an identical budget, calibration regime, and corruption generator. Their numerical results cannot establish rankings or optimal deployment choices. Detailed historical architecture and optimisation metadata, including the recoverable ensemble-member information, are supplied in Supplementary File S1, but a rigorous common-protocol comparison remains necessary. All participants were healthy; neither clinical benefit, incorrect exercise quality assessment, multi-person home conditions, nor long-term drift has been tested. Future work should perform matched multi-seed training of all candidate methods, gather a larger independent-subject cohort, compare multiple datasets and clinically meaningful impairment strata, and prospectively validate the deployment gate before clinical use. 

## Figures and Tables

**Table 1:** Exact mapping from corruption severity *s* to each radar degradation operator used in constrained fine-tuning. Random selections are resampled independently for each sample and corrupted view.

Operator	Numerical Mapping
Frame masking	Zero max(1,round(96s)) randomly selected frames.
Temporal block	Zero one contiguous block of max(1,round(96s)) frames.
Spatial masking	Zero a rectangle of size $\max\left(1,\mathrm{round}\left(14\sqrt{s}\right)\right)\times \max\left(1,\mathrm{round}\left(14\sqrt{s}\right)\right)$ in every frame.
Channel removal	Zero min(5,max(1,⌈5s⌉)) of the five radar feature channels.
Gaussian noise	Add zero-mean Gaussian noise with standard deviation σ=0.04+0.50s in normalised radar units.
Amplitude attenuation	Multiply the radar tensor by a=max(0.15,1−0.90s).
Temporal rolling	Circularly shift the sequence by an integer sampled uniformly from [−m,m], where m=max(1,round(96×0.25s)).

**Table 2:** Radar-only performance across historical operating regimes and the matched final comparison. Historical corruption suites and optimisation protocols differ, so the upper block is descriptive; the lower block is a controlled matched comparison.

Method	Accuracy	Macro-F1	Expected Calibration Error (ECE)	Mean Corrupted Macro-F1	Worst Corrupted Macro-F1
Historical operating-regime results (descriptive only)					
Standard KD	0.675	0.6530	–	–	–
RTPD-Net (historical)	**0.850**	**0.8569**	0.1489	0.5039	0.1702
PARETO-RKD enhanced	0.800	0.7921	0.1580	**0.7229**	**0.6441**
AURORA-RKD ensemble	0.800	0.7927	**0.0889**	0.7110	0.4442
Matched final comparison					
RTPD-Net matched anchor	0.800	0.7902	0.1568	0.5332	0.2737
Constrained RTPD-FT	**0.825**	**0.8106**	**0.1263**	**0.6945**	**0.4584**

Table notation: Boldface identifies the numerically best value within the relevant descriptive or matched block only; it does not denote statistical significance or establish superiority across unmatched historical protocols.

**Table 3:** Fixed-split checkpoint re-evaluation with 11 corruption conditions and five realisations per condition (four independent test subjects). The CI is a subject-cluster bootstrap; exact *p* is a whole-subject swap test. The bootstrap interval does not override the limited exact-test resolution.

Metric	RTPD Anchor	CFT Checkpoint	Difference
Clean accuracy	0.800	0.825	+0.025
Clean macro-F1	0.7902	0.8150	+0.0248
Mean corrupted macro-F1	0.5370	0.6374	+0.1004
Subject-bootstrap 95% CI	–	–	[0.0683, 0.1283]
Exact subject-swap test	–	–	p=0.125

**Table 4:** Provenance of distinct fixed-split evaluation blocks. ‘Means’ refers to macro-F1 averaged over the corresponding corrupted conditions; results are not interchangeable.

Evaluation Block	Models/Checkpoints	RTPD Corrupted	CFT Corrupted	CFT Clean
Original matched fixed split	RTPD anchor vs. selected strong-robust CFT (original run)	0.5332	0.6945	0.8106
Objective ablation	RTPD reference vs. anchor + robust + consistency candidate	0.5452	0.6731	0.8150
Independent seeded re-evaluation	Saved rtpdnet_radar_student.pt vs. checkpoint_strong_robust.pt; five draws/condition	0.5370	0.6374	0.8150

**Table 5:** Objective-level ablation of constrained RTPD fine-tuning. Acceptance was determined exclusively using the predefined validation-set constraints for clean recognition, calibration, and corruption robustness; test-set results were not used for objective selection. We did not subject the RTPD reference to the acceptance gate; it served as the fallback model. Boldface identifies the validation-compliant objective selected for grouped evaluation.

Objective	Validation Decision	Test Macro-F1	ECE	Mean Corrupted Macro-F1	Worst Corrupted Macro-F1
RTPD reference	Reference	0.7902	0.1568	0.5452	0.2331
Clean preservation	Accepted	0.7984	0.1730	0.5282	0.2134
Robust CE only	Rejected	0.8356	0.1106	0.7069	0.5084
Anchor + robust CE	Rejected	0.8169	0.1521	0.5395	0.2826
Anchor + robust + consistency	**Accepted (selected)**	0.8150	0.1444	0.6731	0.4469
Strict clean preserving	Rejected	0.7998	0.1553	0.5378	0.2794

**Table 6:** Five-fold subject-disjoint comparison of the fold-local RTPD-Net reference policy and the validation-gated RTPD/CFT deployment policy. Fine-tuning was deployed in two folds that satisfied the predefined validation constraints; the RTPD-Net reference was retained in the remaining three folds. Values are mean ± standard deviation across the five subject partitions and do not represent repeated initialisation variability.

Policy	Accuracy	Macro-F1	ECE	Mean Corrupted Macro-F1	Fine-Tuning Deployment
Fold-local RTPD-Net (reference)	0.765±0.091	0.7564±0.0896	0.1663±0.0273	0.5602±0.0748	Reference in all 5 folds
Safe constrained deployment	0.780±0.065	0.7712±0.0685	0.1676±0.0286	0.5728±0.0715	FT accepted 2/5; RTPD fallback 3/5

*Table notation:* boldface marks the larger numerical mean within this single five-fold partition; it does not indicate statistical significance or repeated-partition stability.

**Table 7:** Repeated fully fold-local grouped evaluation. Each seed contains five folds and tests all 20 subjects exactly once. “Accepted” counts validation-gated fine-tuning checkpoints among five folds; the remaining folds revert to RTPD. Differences are policy minus anchor in corrupted macro-F1.

Partition Seed	Anchor Corrupted F1	Policy Corrupted F1	Difference	FT Accepted
101	0.5316	0.5565	+0.0249	2/5
202	0.5199	0.5214	+0.0015	2/5
303	0.5641	0.5641	0.0000	0/5
404	0.5736	0.5843	+0.0106	1/5
505	0.4982	0.4982	0.0000	0/5
Five-seed mean	0.5375	0.5449	+0.0074	5/25 (20%)

**Table 8:** All 25 paired fold-level policies minus RTPD-reference differences. “Fallback” denotes a rejected fine-tuning checkpoint, for which both differences are exactly zero by construction. Values are macro-F1 differences, not percentages.

Seed	Fold	Decision	Δ Clean F1	Δ Corrupted F1
101	1	Fallback	+0.0000	+0.0000
101	2	Fallback	+0.0000	+0.0000
101	3	Accepted	+0.0127	+0.1165
101	4	Fallback	+0.0000	+0.0000
101	5	Accepted	+0.0558	+0.0056
202	1	Fallback	+0.0000	+0.0000
202	2	Fallback	+0.0000	+0.0000
202	3	Accepted	-0.0395	+0.0055
202	4	Accepted	+0.0060	+0.0006
202	5	Fallback	+0.0000	+0.0000
303	1	Fallback	+0.0000	+0.0000
303	2	Fallback	+0.0000	+0.0000
303	3	Fallback	+0.0000	+0.0000
303	4	Fallback	+0.0000	+0.0000
303	5	Fallback	+0.0000	+0.0000
404	1	Accepted	+0.0232	+0.0586
404	2	Fallback	+0.0000	+0.0000
404	3	Fallback	+0.0000	+0.0000
404	4	Fallback	+0.0000	+0.0000
404	5	Fallback	+0.0000	+0.0000
505	1	Fallback	+0.0000	+0.0000
505	2	Fallback	+0.0000	+0.0000
505	3	Fallback	+0.0000	+0.0000
505	4	Fallback	+0.0000	+0.0000
505	5	Fallback	+0.0000	+0.0000

## Data Availability

The mRI dataset is publicly available through Dryad at https://doi.org/10.5061/dryad.9ghx3ffpp. The accompanying reproducibility archive contains the executed source notebooks, paired fixed-split predictions, subject/fold split identifiers, acceptance decisions, and seed-wise summary files. Larger checkpoint archives are retained by the authors and can be provided upon request.

## Supplementary Materials

The following supporting information can be downloaded at: https://www.mdpi.com/article/10.3390/s26216970/s1, Supplementary File S1: reproducibility details including exact subject splits, architecture specifications, optimisation settings, corruption operators, objective definitions, and fold-level grouped-subject results.
