# Supplementary material for: Multi-Objective Evaluation of Wearable-Assisted Radar-Only Rehabilitation Monitoring
Source: Sensors (Basel). 2026 Nov 4;26(21):6970. doi: 10.3390/s26216970 (PMC13766377; doi:10.3390/s26216970)
Supplement: Supplementary file 1 [file sensors-26-06970-s001.zip › sensors-4580757-supplementary.pdf]

Supplementary Materials: Multi-Objective Evaluation of Wearable-Assisted Radar-Only Rehabilitation Monitoring

Hrithik Majumdar Shibu [1], Seyed-Ali Sadegh-Zadeh [2] and Delaram Jarchi [1,*]

1. Exact Dataset Protocol

Table S1. Fixed subject-independent split used for the matched experiment.

Partition	Subject identifiers	Segments
Training	03, 04, 05, 06, 08, 09, 11, 12, 13, 17, 18, 20	120
Validation	02, 07, 14, 16	40
Test	01, 10, 15, 19	40

Each subject contributes one segment for each of the ten P2_10 actions. Normalisation statistics are estimated only from training subjects. Each radar sequence is resampled to 96 frames with shape $96 \times 14 \times 14 \times 5$; the 14×14 dimensions form the released radar feature map and the five channels contain x, y, and z coordinate features, Doppler velocity, and return intensity. The six IMUs each provide 12 features (nine orientation-matrix entries and three acceleration values) and are flattened to 96×72.

2. Architecture and Optimisation Details

Table S2. RTPD-Net architecture used as the clean reference and constrained fine-tuning anchor.

Component	Operation	Output / role
Radar frame encoder	Three convolutional stages with batch normalisation, GELU, pooling, and global spatial pooling	128-dimensional token per frame
Radar temporal encoder	Four residual TCN blocks, kernel 3, dilations 1, 2, 4, 8	96 temporal tokens
IMU encoder	Linear projection from 72 features followed by matched temporal refinement	128-dimensional IMU tokens
Teacher fusion	IMU-to-radar cross-attention and reliability-weighted token fusion	Fused teacher tokens and logits
Student pooling	Learned temporal attention pooling	Sequence representation
Student heads	Exercise classifier and gradient-reversed subject classifier during training	10-class radar deployment output
Deployment size	Radar-only RTPD student	Approximately 0.46M parameters

Table S3. Principal optimisation settings.

Stage	Epoch budget	Initial LR	Additional settings
Radar SSL	10	4×10^{-4}	Mask probability 0.30; NT-Xent temperature 0.15
Multimodal teacher	20	3×10^{-4}	AdamW; early stopping; corruption-supervised reliability
RTPD student	22	3×10^{-4}	KD temperature 4; label smoothing 0.03; global gradient L2-norm clipping at 5.0
Constrained FT	10	4×10^{-5}	Anchor temperature 3; validation-constrained checkpointing
Common	–	–	Batch size 24; weight decay 10^{-4}; mixed precision; NVIDIA Tesla P100 GPU

3. PARETO-RKD Reproducibility Specification

PARETO-RKD is an in-house historical method variant developed in this project. Its historical performance is reported descriptively in the main article because its training budget and corruption protocol differ from the final matched RTPD/CFT experiment. The following tables document the archived configuration used to produce that operating-regime result.

Table S4. PARETO-RKD architecture and training stages from the archived implementation.

Component	Configuration	Role
Radar encoder	128-dimensional embeddings; four residual temporal blocks with dilations 1, 2, 4, 8; dropout 0.22	Radar temporal representation
Multimodal teacher	Counterfactual reliability teacher with clean, radar-corrupted, and IMU-corrupted views	Learns reliability-aware multimodal targets
Student	Calibrated radar-only student, 128-dimensional latent representation	Deployment model
Attention / projection	4 attention heads; 96-dimensional projection head	Cross-modal / auxiliary representation processing
Calibration	10 ECE bins; Brier, entropy-match, and temperature-regularisation terms	Calibration-aware training and evaluation
Selection	Validation-only Pareto-safe interpolation grid $\alpha \in \{0, 0.1, \ldots, 1\}$	Retains an anchor when enhanced solution violates tolerances

Table S5. Principal PARETO-RKD optimisation and objective settings.

Setting	Archived configuration
Batch / representation	Batch size 20; embedding dimension 128; projection dimension 96; dropout 0.22; four attention heads.
Optimisation	AdamW; supervised learning rate 3×10^{-4}; SSL learning rate 4×10^{-4}; weight decay 10^{-4}; patience 6; gradient L2-norm clip 5.0; label smoothing 0.03; mixed precision.
Epoch budgets	SSL 10; radar baseline 16; multimodal teacher 22; standard-KD anchor 22; enhanced PARETO student 26.
Knowledge distillation	$T = 4$; standard-KD weight 0.90; adaptive-KD range 0.10–1.10.
Robustness	robust CE weight 0.65; robust KD 0.25; clean–corrupt consistency 0.18; corruption severity 0.08–0.55 with 30% warm-up.
Teacher reliability	gate supervision 0.85; gate ranking 0.45; gate diversity 0.03; teacher expert 0.25; corrupt-teacher 0.60; teacher consistency 0.12; gate-target temperature 0.55.
Gradient-routed auxiliaries	temporal base weight 0.14; relation 0.07; prototype 0.12; auxiliary EMA 0.95; alignment power 2.0; temporal-alignment temperature 0.12; prototype temperature 0.15.
Calibration	Brier weight 0.18; entropy-match 0.08; temperature regularisation 0.015; 10 ECE bins; conformal $\alpha = 0.10$.
Validation safety	clean-F1 tolerance 0.005; robust-F1 tolerance 0.005; ECE tolerance 0.010; NLL tolerance 0.050; interpolation grid $\alpha = 0, 0.1, \ldots, 1.0$.
Primary reproducibility seed	2026.

4. Explicit Historical Layer Dimensions

The following dimensions were verified from the original PARETO-RKD and AURORA-RKD notebook model definitions, rather than inferred from performance. Both methods take 96 radar frames of $14 \times 14 \times 5$ and use three convolutional frame layers: $5 \rightarrow 24$ (3×3), max-pooling 2×2, $24 \rightarrow 48$ (3×3), and $48 \rightarrow 64$ (3×3), followed by global spatial average pooling and a $64 \rightarrow 128$ token projection. For PARETO, the 72-feature IMU token projection is $72 \rightarrow 128$; independent four-block residual TCN refiners use 128 input/output channels and kernel 3 with dilations $1, 2, 4, 8$ (two convolutions per block). Temporal attention pooling applies a LayerNorm(128), linear $128 \rightarrow 64$, Tanh and linear $64 \rightarrow 1$. The main cross-modal attention uses four heads over 128-dimensional tokens and the auxiliary projection dimension is 96.

For AURORA the same frame CNN and 128-dimensional token projection precede the radar temporal options. The compact branch uses four residual TCN blocks (128 channels, kernel 3, dilations $1, 2, 4, 8$). Its hybrid alternative also includes three depthwise temporal convolution kernels $(3, 7, 15)$ with 128 channels followed by individual $128 \rightarrow 128$ pointwise projections, concatenation to 384 channels and a $384 \rightarrow 128$ fusion projection. Its self-attention alternative uses a one-layer Transformer encoder with model dimension 128, four attention heads and feedforward dimension 256. The student classifier operates on the pooled 128-dimensional representation using either a linear ten-class head or a cosine head with scale 16. PARETO radar-student parameters are approximately 454,876; AURORA's reported 4,896,238 describes the sequential eight-member ensemble, not one student. These dimensions describe recoverable implemented blocks, not claims that all optional branches are simultaneously active in every ensemble member.

5. AURORA-RKD Reproducibility Specification

AURORA-RKD is also an in-house historical method variant. It uses a larger candidate library and aggregation stage than RTPD-Net and therefore should not be interpreted as a parameter-matched baseline. The archived configuration is documented here so that its descriptive calibration and robustness results can be reproduced independently.

Table S6. AURORA-RKD architecture and candidate construction.

Component	Configuration	Role
Radar backbone	Multi-resolution radar encoder; 128-dimensional embeddings; TCN dilations 1, 2, 4, 8; dropout 0.22	Multi-scale temporal radar representation
Radar branches	Multi-kernel temporal processing, self-attention, and learned routing	Candidate diversity and complementary temporal context
Teacher	Quality-aware AURORA multimodal teacher with radar/IMU quality gating and hard missing-modality override	Reliability-aware privileged supervision
Attention	4 attention heads	Cross-modal and radar self-attention
Student library	3 seeds × 3 families × 4 training modes = 36 planned candidates	Compact/hybrid linear and cosine-head students
Training modes	standard KD, adaptive KD, robust KD, robust group-KD	Candidate operating regimes
Aggregation	Validation-selected non-negative ensemble, at most 8 candidates, 5000 search draws	Post-hoc aggregation without negative weights

Table S7. Principal AURORA-RKD optimisation and objective settings.

Setting	Archived configuration
Batch / representation	Batch size 20; embedding dimension 128; TCN channels 128; dilations 1, 2, 4, 8; dropout 0.22; four attention heads; cosine scale 16.0.
Optimisation	AdamW; supervised learning rate 3×10^{-4}; SSL learning rate 4×10^{-4}; weight decay 10^{-4}; patience 6; gradient L2-norm clip 5.0; label smoothing 0.03; mixed precision.
Epoch budgets	SSL 10; quality-aware teacher 24; each student candidate 20.
Candidate library	Student seeds 2026, 2027, 2028; families `compact_linear`, `hybrid_linear`, and `hybrid_cosine`; modes standard KD, adaptive KD, robust KD, and robust group-KD (36 planned combinations).
Knowledge distillation	$T = 4$; KD weight 0.90; adaptive-KD range 0.10–1.25.
Robustness	robust loss weight 0.55; robust KD 0.20; consistency 0.16; AugMix 0.20; severity 0.08–0.55 with 35% warm-up.
Group robustness	group-DRO temperature 4.0 and weight 0.30; supervised-contrastive weight 0.06 and temperature 0.12.
Teacher quality objectives	quality 0.75; gate target 0.70; gate direction 0.55; expert 0.30; corrupt-teacher 0.65; teacher consistency 0.12; quality-target temperature 0.55.
SWA / aggregation	SWA enabled from 65% of training at LR 8×10^{-5}; non-negative ensemble uses at most 8 candidates and 5000 validation search draws.
Validation / calibration	clean-F1 tolerance 0.005; robust-F1 tolerance 0.005; ECE tolerance 0.015; 10 ECE bins; conformal $\alpha = 0.10$.
Primary reproducibility seed	2026; candidate-library seeds listed above.

6. Historical-Method Splits and Ensemble Composition

Both executed historical PARETO-RKD and AURORA-RKD notebooks report the same fixed subject identifiers: training = 03, 04, 05, 06, 08, 09, 11, 12, 13, 17, 18, 20; validation = 02, 07, 14, 16; test = 01, 10, 15, 19. This equality of subject IDs does *not* make their results matched: the optimisers, random seeds, model-selection procedures, training budgets and some test corruption conditions differ. The executed PARETO-RKD robustness list contains ten conditions (three frame masks, temporal block 0.30, two channel drops, two Gaussian strengths, spatial mask 0.30, amplitude 0.50) excluding temporal roll; the AURORA-RKD list adds temporal roll 0.50. Their robustness evaluation severities are 0.10/0.30/0.50 for frame loss, 0.30 for block loss, 0.20/0.40 for channel loss, 0.25/0.50 for Gaussian noise, 0.30 for spatial loss and 0.50 for amplitude loss. These are test perturbation settings, distinct from the training corruption curricula (PARETO 0.08–0.55; AURORA 0.08–0.55).

The archived PARETO radar-only student has approximately 454,876 parameters. The original AURORA evaluation reports approximately 4,896,238 parameters when all ensemble members are counted sequentially; this is *not* a single radar-student parameter

count. The executed AURORA notebook recovers the following eight selected candidate identities and non-negative weights, retained here with source precision.

Table S8. AURORA-RKD ensemble membership recovered from the executed historical notebook. These weights are the historical selected candidate weights and must not be interpreted as the output of a matched comparison.

Candidate identity	Validation-selected weight
`hybrid_cosine__robust_kd__seed2027`	0.5480369
`hybrid_cosine__robust_kd__seed2028`	0.2768356
`hybrid_linear__robust_kd__seed2028`	0.1482151
`hybrid_cosine__robust_group_kd__seed2027`	0.0165001
`compact_linear__standard_kd__seed2026`	0.0085692
`hybrid_cosine__robust_group_kd__seed2028`	0.0017921
`compact_linear__robust_kd__seed2027`	0.0000502
`hybrid_linear__robust_kd__seed2027`	0.0000006

7. Radar Corruption Operators

Table S9. Corruption operators used for robust training and evaluation.

Operator	Definition	Deployment interpretation
Frame mask	Randomly set a specified fraction of frames to zero	Packet/frame loss
Temporal block	Remove one contiguous temporal interval	Burst loss or temporary occlusion
Spatial mask	Zero a rectangular region of each radar frame	Reduced spatial visibility
Channel drop	Remove selected radar channels	Receiver/channel failure
Gaussian noise	Add zero-mean Gaussian perturbation	Measurement noise / interference
Amplitude attenuation	Multiply the radar tensor by a reduced scale	Weak return or range-dependent attenuation
Temporal roll	Circularly shift the frame sequence	Timing and synchronisation disturbance

The fixed matched suite includes frame masking at 10, 30, and 50%; a 30% temporal block; channel dropping at 20 and 40%; Gaussian noise at 0.25 and 0.50 severity; 30% spatial masking; 50% amplitude attenuation; and 50% temporal rolling severity.

Table S10. Exact numerical mapping from the constrained-fine-tuning severity variable s to each radar corruption. Here $T = 96$, $H = W = 14$, and $C = 5$.

Operator	Implementation at severity s
Frame mask	Set $\max(1, \mathrm{round}(Ts))$ independently selected time steps to zero.
Temporal block	Set one contiguous block of $\max(1, \mathrm{round}(Ts))$ time steps to zero.
Spatial mask	Set one rectangular region of size $\max(1, \mathrm{round}(H\sqrt{s})) \times \max(1, \mathrm{round}(W\sqrt{s}))$ to zero across all time steps and channels.
Channel drop	Set $\min(C, \max(1, \lceil Cs \rceil))$ randomly selected radar channels to zero.
Gaussian noise	Add i.i.d. zero-mean Gaussian noise with standard deviation $0.04 + 0.50s$ in normalised radar units.
Amplitude attenuation	Multiply the complete radar tensor by $\max(0.15, 1 - 0.90s)$.
Temporal roll	Circularly shift by an integer chosen uniformly from $[-m, m]$, with $m = \max(1, \mathrm{round}(0.25Ts))$.

8. RTPD Equal-Budget Component Ablation

The following archived 12-epoch ablation isolates the cumulative addition of RTPD auxiliary objectives under a common budget. It is an internal component analysis rather than a matched comparison against PARETO-RKD or AURORA-RKD.

Table S11. Equal-budget RTPD component ablation on the fixed split. The results show that auxiliary terms are not monotonically beneficial under this short common training budget.

Training objective	Test accuracy	Test macro-F1
Hard labels only	0.625	0.5783
Standard logit KD	0.775	**0.7825**
Logit KD + temporal alignment	0.700	0.6921
+ relation matching	0.700	0.6749
+ prototype supervision	0.675	0.6535
Complete RTPD objective (incl. subject adversary)	0.750	0.7394

The non-monotonic pattern is reported explicitly because it limits causal claims about individual RTPD terms. It also motivates the later constrained fine-tuning experiment, which targets robustness without adding another deployment architecture.

9. Constrained Fine-Tuning Objective Ablation

Table S12. Full objective-level ablation. Objective selection used validation constraints only; test-set results were not used for objective selection, and the RTPD reference served as the fallback model.

Variant	Validation decision	Val. clean macro-F1	Val. mean corrupted macro-F1	Test clean macro-F1	Test mean corrupted macro-F1	Test worst corrupted macro-F1
RTPD reference	Reference	0.7686	0.4665	0.7902	0.5452	0.2331
Clean preservation	Accepted	0.7695	0.4950	0.7984	0.5282	0.2134
Robust CE only	Rejected	0.7971	0.7277	0.8356	0.7069	0.5084
Anchor + robust CE	Rejected	0.7433	0.5178	0.8169	0.5395	0.2826
Anchor + robust + consistency	Accepted; selected	0.7667	0.6756	0.8150	0.6731	0.4469
Strict clean preserving	Rejected	0.7424	0.5386	0.7998	0.5378	0.2794

Robust-CE-only obtained the strongest test result but failed the predefined validation conditions. It was therefore excluded from grouped evaluation. This distinction is central to the anti-cherry-picking design.

10. Validation-Gate Sensitivity Scope

The gate uses four criteria: clean macro-F1 decrease ≤ 0.005, clean accuracy decrease ≤ 0.01, ECE increase ≤ 0.03, and a positive mean-corrupted macro-F1 gain. The archived objective-ablation summaries allow a limited audit of the clean macro-F1 criterion. The selected anchor+robust+consistency candidate has validation clean macro-F1 0.7667 versus 0.7686 for the RTPD anchor, a deficit of 0.0019; it therefore remains eligible under clean-F1 tolerances of 0.002, 0.005, and 0.010. The anchor+robust-CE (0.7433) and strict-clean-preserving (0.7424) candidates remain outside even a 0.010 tolerance. A complete multi-dimensional sensitivity analysis cannot be reconstructed from the archived summaries because all per-epoch validation accuracy and ECE values were not retained. Accordingly, the reported 2/5 grouped-fold acceptance rate is conditional on the specified thresholds and composite-score weights and is not claimed to be threshold invariant.

11. Fold-Level Subject-Disjoint Results

Table S13. Fold-level clean and corrupted performance. The constrained row equals RTPD when validation rejected fine-tuning.

Fold	Policy	Accepted FT	Accuracy	Macro-F1	Mean corrupted macro-F1	Worst corrupted macro-F1
1	RTPD reference	–	0.625	0.6277	0.4811	0.2301
1	Safe constrained deployment	Yes	0.700	0.6973	0.5153	0.2964
2	RTPD reference	–	0.725	0.7060	0.4767	0.1566
2	Safe constrained deployment	No; fallback	0.725	0.7060	0.4767	0.1566
3	RTPD reference	–	0.825	0.8271	0.6268	0.3290
3	Safe constrained deployment	No; fallback	0.825	0.8271	0.6268	0.3290
4	RTPD reference	–	0.800	0.7778	0.6170	0.2631
4	Safe constrained deployment	No; fallback	0.800	0.7778	0.6170	0.2631
5	RTPD reference	–	0.850	0.8433	0.5992	0.1183
5	Safe constrained deployment	Yes	0.850	0.8479	0.6281	0.1214

Table S14. Grouped-subject mean ± standard deviation across the five subject-disjoint folds.

Policy	Accuracy	Macro-F1	ECE	Mean corrupted macro-F1	Worst corrupted macro-F1
RTPD reference	0.765 ± 0.0912	0.7564 ± 0.0896	0.1663 ± 0.0273	0.5602 ± 0.0748	0.2194 ± 0.0840
Safe constrained deployment	0.780 ± 0.0647	0.7712 ± 0.0685	0.1676 ± 0.0286	0.5728 ± 0.0715	0.2333 ± 0.0901

12. Seeded Paired Corruption Evaluation

The fixed held-out subjects are 01, 10, 15 and 19. Two saved model state dictionaries were reloaded and evaluated on the same normalised radar tensors for each corruption realisation. A total of 40 clean and 2,200 corrupted paired segment predictions were archived, including logits, probabilities, subject IDs, labels, corruption types and random seeds. The five realisations per condition are *not* additional independent subjects. The 11 conditions below exclude clean radar. The source files `C1_condition_means_checked.csv` and `C1_subject_descriptive_deltas.csv` are included with the supporting experiment files.

Table S15. Five-realisation condition-level mean macro-F1 from the seeded fixed-split checkpoint evaluation. Values are averaged over the five seeded realisations; they are not a subject-independent significance test.

Corruption condition	RTPD	CFT	Difference
Frame mask 10%	0.7915	0.8063	+0.0149
Frame mask 30%	0.4859	0.6820	+0.1960
Frame mask 50%	0.3145	0.3929	+0.0784
Temporal block 30%	0.2548	0.8158	+0.5611
Channel removal 20%	0.5446	0.5962	+0.0516
Channel removal 40%	0.3353	0.4253	+0.0900
Gaussian severity 0.25	0.8271	0.8039	-0.0232
Gaussian severity 0.50	0.7597	0.7662	+0.0065
Spatial mask 30%	0.4700	0.5386	+0.0686
Amplitude attenuation 50%	0.3327	0.3678	+0.0351
Temporal roll severity 0.50	0.7912	0.8164	+0.0252
**Mean across conditions**	**0.5370**	**0.6374**	**+0.1004**

The exact two-sided paired subject-model-swap test returns $p = 0.125$ because only 2^4 subject-level assignments are possible. The subject-bootstrap 95% percentile interval [0.0683, 0.1283] is conditional on four observed subjects and must be interpreted cautiously. The original one-realisation study reported a larger corrupted gain (+0.1613); the saved checkpoint and independent corruption realisations do not yield that exact figure.

13. Five-Seed Fully Fold-Local Repeated Evaluation

The five partition seeds are 101, 202, 303, 404 and 505. Each seed has five complete 12/4/4 subject-disjoint train/validation/test folds, and each of the 20 subjects is tested exactly once per seed. Models and normalisation are retrained inside every fold. Paired test results use 11 corrupted conditions and three realisations per condition. The corresponding source files `C2_25_fold_summary_checked.csv` and `C2_five_seed_summary_checked.csv` provide exact fold means, validation decisions and test subject lists, without rounding.

Table S16. Five-seed aggregate clean and corrupted macro-F1 for the reference and validation-gated policy. "Accept" is the count of validation-gated fine-tunings in five folds.

Seed	Clean reference	Clean policy	Corrupted reference	Corrupted policy	Accept
101	0.7049	0.7176	0.5316	0.5565	2/5
202	0.6962	0.6938	0.5199	0.5214	2/5
303	0.7680	0.7680	0.5641	0.5641	0/5
404	0.7548	0.7589	0.5736	0.5843	1/5
505	0.6705	0.6705	0.4982	0.4982	0/5
**Mean**	**0.7189**	**0.7218**	**0.5375**	**0.5449**	**5/25**

The seed-level bootstrap (resampling five seed means, not individual folds) returns [0.0003, 0.0170] for the corrupted policy–reference difference (+0.0074), and [-0.0010, 0.0076] for the clean macro-F1 difference (+0.0029). Since all seeds reuse the same 20 subjects, these are conditional partition-sensitivity intervals, not independent-cohort confidence intervals. The acceptance rate is only 20% (5/25 folds), with 0/5 accepted in two of five partition seeds. The preceding tables report the single-partition estimates separately from these repeated-seed results.

14. Complete 25-Fold Paired Policy Differences

Table S17. Fold-level differences: validation decision, clean and corrupted macro-F1, gated policy minus fold-local anchor. Rejected updates are anchor fallbacks.

Seed	Fold	Decision	Δ clean	Δ corrupted
101	1	Fallback	+0.0000	+0.0000
101	2	Fallback	+0.0000	+0.0000
101	3	Accepted	+0.0127	+0.1165
101	4	Fallback	+0.0000	+0.0000
101	5	Accepted	+0.0558	+0.0056
202	1	Fallback	+0.0000	+0.0000
202	2	Fallback	+0.0000	+0.0000
202	3	Accepted	-0.0395	+0.0055
202	4	Accepted	+0.0060	+0.0006
202	5	Fallback	+0.0000	+0.0000
303	1	Fallback	+0.0000	+0.0000
303	2	Fallback	+0.0000	+0.0000
303	3	Fallback	+0.0000	+0.0000
303	4	Fallback	+0.0000	+0.0000
303	5	Fallback	+0.0000	+0.0000
404	1	Accepted	+0.0232	+0.0586
404	2	Fallback	+0.0000	+0.0000
404	3	Fallback	+0.0000	+0.0000
404	4	Fallback	+0.0000	+0.0000

Seed	Fold	Decision	Δ clean	Δ corrupted
404	5	Fallback	+0.0000	+0.0000
505	1	Fallback	+0.0000	+0.0000
505	2	Fallback	+0.0000	+0.0000
505	3	Fallback	+0.0000	+0.0000
505	4	Fallback	+0.0000	+0.0000
505	5	Fallback	+0.0000	+0.0000

15. Reproducibility and Claim Boundaries

- Historical RTPD-Net, PARETO-RKD, and AURORA-RKD values are used to identify operating regimes. They are not presented as a matched statistical leaderboard because budgets, seeds, and some corruption definitions differ. Their archived architecture and optimisation settings are documented above for reproducibility, but a future common-protocol retraining is still required for a fair inferential comparison.
- The original RTPD-versus-CFT comparison is matched within its own run. A separate checkpoint re-evaluation is matched within each paired corruption realisation, but does not reproduce precisely the original reported 0.6945 corruption score.
- Grouped folds recompute training statistics and retrain SSL, the teacher, student, calibration, and fine-tuning pipeline. No fold test subject contributes to training or checkpoint selection. The repeated analysis includes five partition seeds (25 complete folds); as all seeds reuse the same 20 participants, the resulting variation is conditional on the participant cohort.
- The study evaluates healthy-subject exercise recognition. It does not establish clinical effectiveness, exercise-quality scoring, or patient rehabilitation outcomes.
